# Supplementary material for: Polydextrose reduces the infection of Klebsiella pneumoniae in mice by downregulating the expression of TamA
Source: Microbiol Spectr. 2025 Oct 1;13(11):e01017-25. doi: 10.1128/spectrum.01017-25 (PMC12584633; doi:10.1128/spectrum.01017-25)

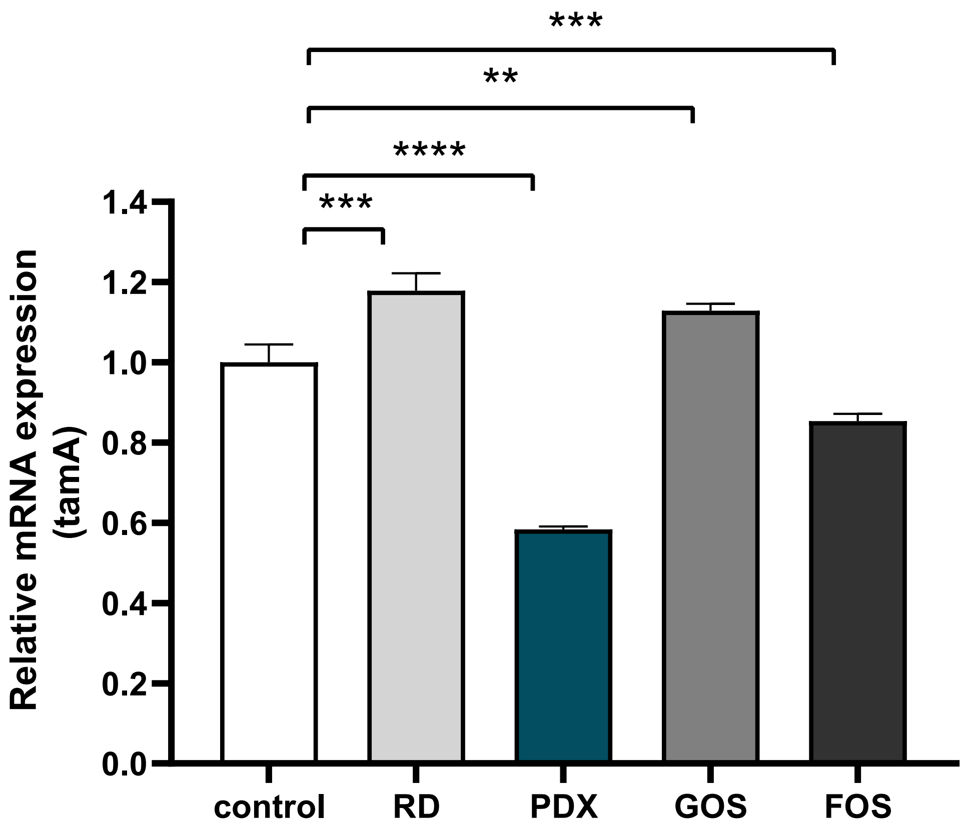

Supplemental Figure S1. The effect of different prebiotics on the *tamA* expression of *K. pneumoniae*. RD: Resistant dextrin, PDX: polydextrose, GOS: galactooligosaccharides, and FOS: fructooligosaccharides. Significance was assessed using a one-way ANOVA test and t-test, followed by Tukey's post-hoc test for multiple comparisons. *P<0.05; **P<0.01; ***P<0.001; ****P<0.0001;

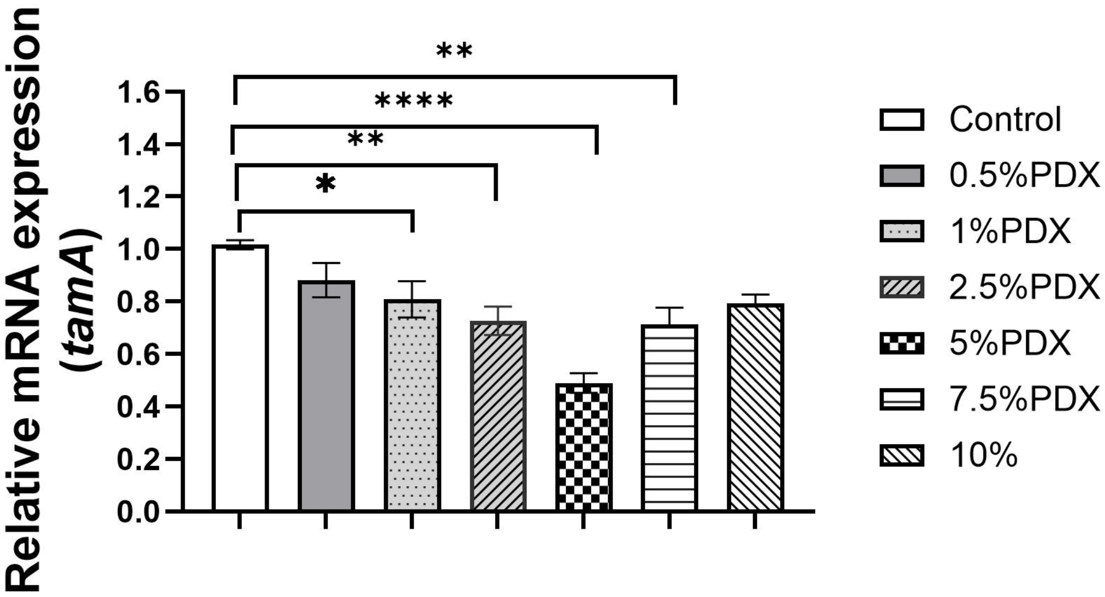

Supplemental Figure S2. The effect of different concentrations of PDX on the *tamA* expression of *K. pneumoniae*. PDX: polydextrose. Significance was assessed using an unpaired t-test followed by Tukey's post-hoc test for multiple comparisons. *P<0.05; **P<0.01; ***P<0.001; ****P<0.0001;

Supplemental Figure S3- WB original images

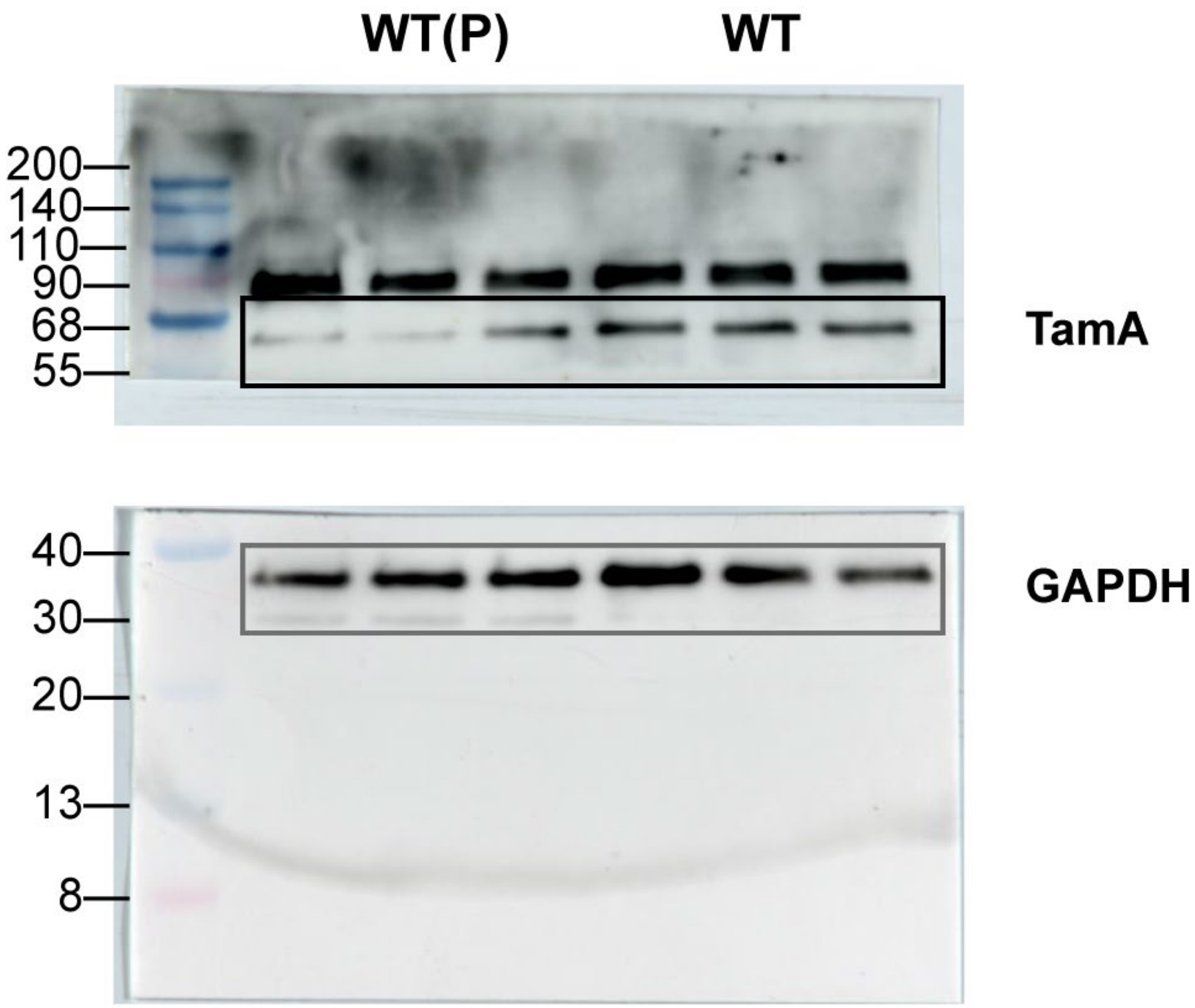

Supplement: Supplemental material — Fig. S1 to S3. [file spectrum.01017-25-s0001.pdf]
